# Bright-field Nanoscopy: Visualizing Nano-structures with Localized Optical Contrast Using a Conventional Microscope

**DOI:** 10.1038/srep25011

**Published:** 2016-04-26

**Authors:** Swathi Suran, Krishna Bharadwaj, Srinivasan Raghavan, Manoj M. Varma

**Affiliations:** 1 Center for Nano Science and Engineering (CeNSE), Indian Institute of Science, Bangalore, INDIA 560012; 2 Electrical Communication Engineering, Indian Institute of Science, Bangalore, INDIA 560012.

## Abstract

Most methods for optical visualization beyond the diffraction limit rely on fluorescence emission by molecular tags. Here, we report a method for visualization of nanostructures down to a few nanometers using a conventional bright-field microscope without requiring additional molecular tags such as fluorophores. The technique, Bright-field Nanoscopy, is based on the strong thickness dependent color of ultra-thin germanium on an optically thick gold film. We demonstrate the visualization of grain boundaries in chemical vapour deposited single layer graphene and the detection of single 40 nm Ag nanoparticles. We estimate a size detection limit of about 2 nm using this technique. In addition to visualizing nano-structures, this technique can be used to probe fluid phenomena at the nanoscale, such as transport through 2D membranes. We estimated the water transport rate through a 1 nm thick polymer film using this technique, as an illustration. Further, the technique can also be extended to study the transport of specific ions in the solution. It is anticipated that this technique will find use in applications ranging from single-nanoparticles resolved sensing to studying nanoscale fluid-solid interface phenomena.

Visualization of structures below the optical diffraction limit requires special super-resolution techniques which are now well developed in the case of fluorescence imaging. Such techniques include Stimulated Emission Depletion (STED)^1^, Stochastic Optical Reconstruction Microscopy (STORM)^2^ and Photo-activated Localization Microscopy (PALM)^3^. In many situations one may only need to detect the presence of a nanoscale feature without needing to resolve it. For instance, the ability to count single nanoparticles may be of use in studying stochastic protein-protein interactions at the few-molecule limit^4^. We use the term visualization to refer to the detection of spatial features without necessarily being able to optically resolve the detailed geometry of the feature. Among the far-field, non-fluorescent techniques used for visualizing nano-scale features, dark-field (DF) microscopy can detect single metal nanoparticles down to 40 nm based on the Localized Surface Plasmon Resonance (LSPR) effect^5^. However DF microscopy requires much larger sizes with dielectric particles. Photothermal imaging (PHI)^6^ can detect objects down to 1.4 nm but requires intense pump sources^7^. Spatial Modulation Spectroscopy (SMS)^8^ can visualize nanoscale objects by using phase locked detection of the intensity modulation caused by the motion of the nanoscale object moving in and out of the focal volume. Detection of 50 nm metal particles was demonstrated using SMS and the limit of detection was projected to be around 5 nm. Recently, it was shown that scattering from single protein molecules can be obtained under optimal signal-to-noise conditions achieved through control of the illumination and detection parameters^9^. Here, we present a technique to visualize nanoscale surface features based on the optical contrast generated by the differential etching of a germanium (Ge) thin film (~30 nm thick) deposited on gold in the presence of water. Such ultra-thin Ge films on gold produce a strong thickness dependent color response^10^. These Ge films also get etched in water due to the dissolution of its oxide GeO_2_ in water. Any nanoscale structure placed on the Ge film, for instance nanoparticles dispersed on the film surface, impedes the transport of water to the Ge film resulting in a differential transverse etch rate and consequently a local color difference. The lateral etching of the underlying Ge film further amplifies the dimensions of the feature/structure allowing the local contrast to be observed using a conventional microscope with bright-field illumination. This technique works for applications ranging from visualisation of single nanoparticles to extended nanostructures such as graphene grain boundaries and defect lines as thin as 2–5 nm. There has been significant recent interest in using graphene and other 2D materials as desalination^11^ and filtration membranes^12^,^13^,^14^. Our technique can provide high resolution maps of water transport through such nano-membranes. This is possible by measuring the optical contrast between the nano-membrane and the background as a function of time, discussed in detail in further sections. The rate of change of optical contrast provides a measure of local water transport rates across the nano-membrane. We demonstrate this ability by measuring the volume rate of transport of water across a 1 nm thick polymer film deposited on top of the Ge layer. The technique can also be extended to probing the transport of ions in an aqueous solution. Also, anisotropic etch rates have been reported for metal nanoparticles deposited on crystalline Ge (100 orientation) in the presence of water^15^. The metal nanoparticles catalyze the oxidation of Ge to GeO_2_ in their neighbourhood leading to local enhancement of etch rates. We have observed a similar enhancement of lateral etch rates in our films in the presence of metal (Ag) nanoparticles (40 nm dia.). Based on this data, we estimate that this technique should enable the visualization of single nanoparticles down to about 2 nm.

## Mechanism of visualization

Anti-reflective (AR) coatings are necessary in several applications where losses from reflection of incident optical radiation need to be eliminated^16^. Reflective losses from metallic surfaces can be eliminated by depositing ultra-thin films of absorbing dielectrics on the metal surface^17^,^18^,^19^. Mikhail *et al*.^10^ used ultra-thin Ge films (5–25 nm thick) deposited on gold to achieve anti-reflection condition for enhancing solar energy conversion efficiency. Germanium readily forms an oxide GeO_2_, which dissolves in water^15^ leading to progressive reduction of the Ge film thickness upon immersion in water. Concomitant with the reduction of Ge film thickness, the AR condition shifts leading to a change in color of the film, as described in^10^. Our device consists of a 30 nm thick Ge film deposited on an optically thick gold film on a Silicon substrate. Any micro- or nano-scale structure on such a device produces a local difference in the water transport rate which translates to a local color difference due to differential transverse etch rates, [See Fig. S4 in Supplementary Information (SI) text, Section 5]. These thickness differences are significant enough to produce local color contrast differences due to thin film interference. A simultaneous lateral etching of the Ge film amplifies the lateral dimensions of the nanoscale feature making the color contrast observable under a regular bright-field microscope without any external modifications. This optical contrast can be enhanced using Differential Interference Contrast (DIC) mode as seen in the results section.

## Results

**Figure 1:**
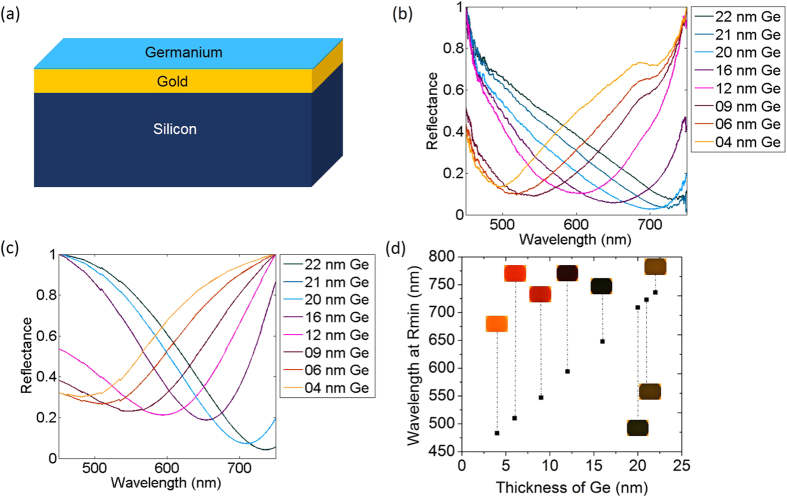
Experimental and calculated reflectance spectra of the device. (**a**) Schematic of the device. (**b**) The normalized experimental reflectance spectra for different thicknesses of Ge on Au (**c**). The calculated reflectance spectra for the same thicknesses of Ge on Au. (**d**) Corresponding reflectance minimum plotted for different thicknesses of Ge. The Bright-field microscope images as a function of Ge thickness are inserted next to each plotted data point. All images are captured at 50× magnification.

We fabricated devices with Ge thicknesses ranging from about 5 nm to 30 nm to extensively characterize their color response and etch rates in water. The details of this characterization effort are provided in the Methods section and SI text, sections 1, 2 & 4. Figure 1(b) shows the experimentally measured shift in the reflectance spectra of one such device as the Ge film progressively got etched in water. Bright-field images as well as reflectance spectra were simultaneously acquired using a 63× water immersion objective. The experimental data matched well with the expected spectra (Fig. 1(c)) computed using a transfer-matrix method [SI text, section 3]. The experimental and computed reflectance spectra were respectively normalized to a maximum value of one. The shift of the reflectance spectra manifests as a change in color of the device (Fig. 1(d)). It is also seen from Fig. 1(d) that the position of the reflectance minimum red-shifts nearly linearly with increasing Ge film thickness. The data for our devices are consistent with previous reports^10^. It is evident that a thickness change of a few nm can produce appreciable color change.

We investigated if defects and grain boundaries in CVD grown single layer graphene (CVD-SLG) can be visualized using this technique. While large morphological features like tears and folds of graphene are readily visible under a simple optical microscope, visualising structures such as grain boundaries and pinhole defects in graphene requires High Resolution Transmission Electron Microscope (HR-TEM)^20^ and Scanning Transmission Electron Microscope (STEM)^21^,^22^. We were motivated by the expectation that Ge film directly underneath a defect may etch at a faster rate due to possibly higher rate of water transport through the defects relative to the non-defective area. The resultant color contrast would allow us to conveniently identify graphene grain boundaries (GGBs) and other defects using a regular optical microscope instead of an HR-TEM or STEM. In order to test this hypothesis, a large area graphene film was initially synthesised on a Copper foil in a CVD chamber and subsequently transferred onto our device. The details of the growth conditions and transfer procedure are described in the methods section. Raman measurements confirmed the presence of single layer graphene on the device [SI text, section 7].

**Figure 2:**
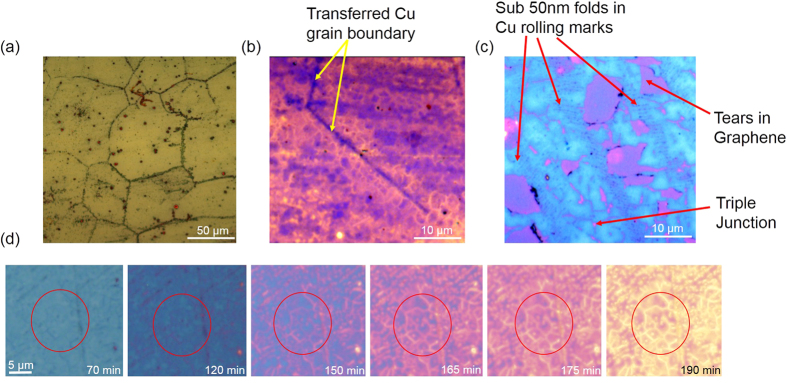
Observation of defects in graphene. (**a**) A bright-field image showing defects in graphene along the Cu grain boundaries. (**b**) Observation of grain boundaries in Graphene along the Cu grain boundary (indicated with yellow arrows). This Bright-field (BF) image has been post processed to improve visualization. The boundaries encircling grains (GGBs) appear yellow in the image. We clearly see that the typical grain sizes are about 2–5 μms. (**c**) A post processed BF image where GGBs, tears in graphene, triple junction and folds along the Cu rolling marks are clearly visible. The Ge beneath the GBs/other defects etch faster (pinkish region) than at the grains themselves (large blue regions). (**d**) Shows the evolution of the appearance of GGBs with time. The raw images of (**b,c**) are shown in the [SI text, section 8].

About 20 μL of deionised water (DI water) was drop-casted on the transferred graphene film, covering it entirely. We then used a 63× water immersion objective to observe the time-course of water transport through these graphene films. As time progressed, an etching pattern, shown in Fig. 2(a), representing a grain size of about 50 μm became evident at first in the Ge film. The average grain size of our graphene monolayer is of the order of 2–5 μm [SI text, section 7] whereas the grain size of the Cu foils on which the monolayers are grown have a grain size of 50 μm. Therefore, the 50 μm scale features observable initially are presumably due to the transfer of Cu grain boundary pattern onto the graphene monolayer which is grown on top. These high energy boundaries on the Cu surface are responsible for many more nucleation sites for graphene grains and therefore a large density of graphene grain boundaries per unit area is expected around them^23^. The graphene monolayer is thus expected to be more defective in these regions. Our data thus reveals two aspects about the graphene monolayers. One, the grain boundaries in the Cu translate to defects in the graphene monolayer and two, these boundaries are more permeable to water. After approximately 2 hrs of etching, the GGBs (2–5 nm in width) were clearly visible (Fig. 2(b,d)) with a noticeable contrast due to transverse and lateral etching of the underlying Ge as discussed in the previous sections. The tears in the film, folds in Graphene along the Cu rolling marks etc. can also be clearly distinguished (Fig. 2(c)). The color balance of Fig. 2(b,c) have been adjusted for greater visual contrast using open source software ImageJ^24^. The raw images of 2(b,c) are shown in the SI text, section 8. The complete time course of the etching process is presented as supplementary video 1. The differential etching observed here is likely to be a combination of enhanced oxidation^25^ and subsequent dissolution of the Ge film underneath defective areas of graphene.

**Figure 3:**
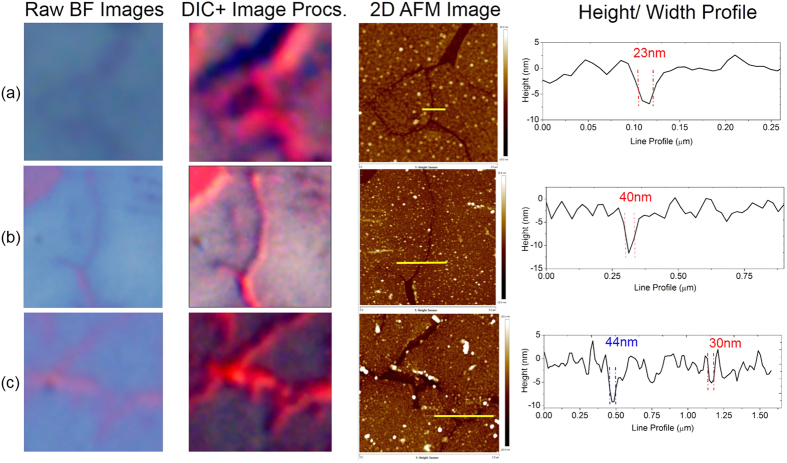
Imaging grain boundaries (GB) from different SLGs. (**a**) GBs visualized in SLG grown with low defect densities. First column shows raw image obtained from Bright-field (BF) mode in an optical microscope, the second column contains the color balance adjusted version of the images obtained using the DIC mode. The third and the fourth column shows the corresponding AFM data, a 2D AFM scan and the height profile measuring the width of the etched Ge trench across the solid yellow line marked in the AFM image. (**b**,**c**) Show visualization of GBs in SLGs grown with higher defect densities in BF and DIC mode with the AFM data supporting it. The optical images are cropped and magnified from a 100× image taken in both BF and DIC modes. The raw BF and DIC images are shown in the SI text, section 8.

GGBs allow lateral etching of the underlying Ge thin film, amplifies the true widths (2–5 nm) of GBs to trenches in Ge of 20 nm in width as measured in AFM, shown in Fig. 3. The ability to detect features of dimensions about 2–5 nm using an optical microscope, points to the potential of this technique for nanoscopic applications. Raman measurements performed after the etching process confirmed the presence of an intact single layer graphene film post-water etch [SI text, section 7]. In addition to features on single layer graphene, we were also able to visualize other nanoscale features such as photoresist lines [Data not shown] thus demonstrating the general applicability of this technique.

**Figure 4:**
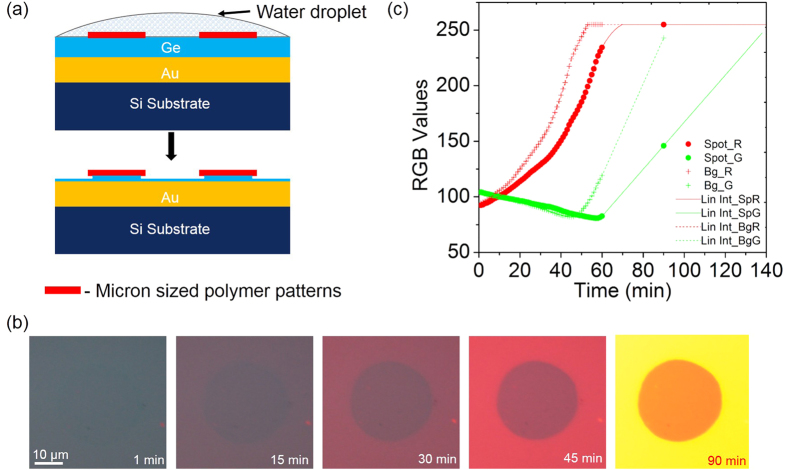
Differential water transport through nanometer thick polymer films. (**a**) Schematic showing polymer films patterned (spot) on the Ge device undergoing etching in the presence of a water droplet. After 90 mins of etch, Ge at the background has almost completely eroded when compared to the Ge beneath the polymer. (**b**) Sequence of images showing as Ge etched at the polymer pattern (spot) and the background (Bg). All optical images have been captured using a 63× water immersion objective. The time stamp is marked in each of these images indicating when the image was taken with respect to the first image. (**c**) A plot of R, G and B obtained over time from the spot and background (bare Ge) is compared to that of the bare Au deposited.

In order to investigate the use of this technique to measure water transport through ultra-thin (1–5 nm thick) membranes (referred to as nano-membranes in this article), we fabricated a microarray pattern of a single layer of a polyelectrolyte^26^, PAH (Poly(allylamine Hydrochloride)) on top of our device as shown in Fig. 4(a). The thickness of a single layer of PAH is about 1 nm [SI text, section 9]. The time course of water etching of this device with the PAH pattern was measured using the 63× water immersion objective. Initially, the polymer microarray was not visible against the background (Ge device) due to low optical contrast as seen in Fig. 4(b). However due to the difference in the rate of water transport through the polymer nano-membrane relative to the background (bare Ge) the optical contrast gradually increases due to the increasing difference in the underlying Ge film thickness. The complete sequence of etching is provided as supplementary video 2. The rate of change of color (or more precisely, the spectral position of the reflectance minimum) of the region containing the polymer, relative to the background is a measure of the transport rate of water through the polymer. For e.g., if water can pass unimpeded through the membrane, we would expect the rate of color change between the polymer and the background to be the same. On the other hand, if the polymer offers high resistance to the transport of water, we would expect no change in the color of the region containing the polymer while there would be a gradual change in the background color until it reaches the gold film which acts as an etch stop. In principle, measurement of color differences can enable the extraction of transport rates of water in these systems. In practice, precise quantification of transport rates using color values is challenging due to a) non-linear behaviour of color values with respect to spectral shifts and b) a detailed quantitative model to relate Ge etch rate differences between the region under the membrane and the background. It is possible that different membranes in addition to offering varying resistance to water transport also change the permeability of oxygen which would affect the GeO_2_ content under the membrane. It is GeO_2_ which dissolves in water and not Ge directly. A precise quantitative estimation must take care of these effects rigorously. Although at present, we do not have such a rigorous model, a naive model [SI text, section 9] yields a transport rate of 510 pL/min through the 1 nm thick PAH layer. Using appropriate quantitative models, this technique may be useful for fundamental studies of water transport through nano-scale membranes^12^,^13^,^14^ as well as high resolution mapping of water transport heterogeneity in such membranes. As described later in the article, this technique could also be extended to measure the transport of ions through nano-membranes using selective intermediate polymer layers between the nano-membrane and the Ge film.

**Figure 5:**
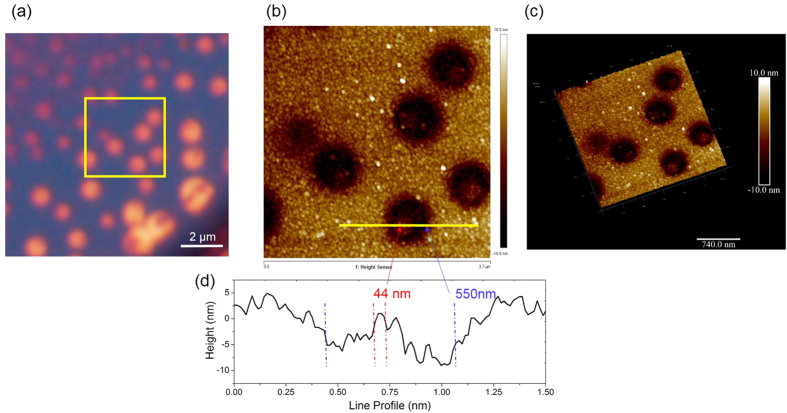
Visualizing 40 nm Ag NPs. (**a**) Optical image showing NPs which caused the substrate, Ge to etch laterally around the particle to form circular pits. (**b**) Shows the AFM of the area in the yellow box in (**a**). (**c**,**d**) are the 3D view and line profile along line marked in (**b**).

As a final demonstration of the utility of this technique in nanoscopy, we imaged isolated metal nanoparticles using this technique. To ensure adhesion of the particle to the Ge film during the rather long etching process (~90 minutes), we used triangular and disk shaped Ag nanoparticles with size ~40 nm [SI text, section 10]. We hypothesized that these NPs with larger surface area of contact would adhere to the Ge layer without the use of additional functionalization layers. It has been shown earlier that metal NPs can lead to enhanced etch rate of Ge (100 orientation) crystal planes due to the catalytic action of the metal NPs. This phenomenon led to a lateral expansion of the feature size as revealed by electron microscopy^15^. Our work deals with Ge thin films as opposed to crystalline Ge wafers. Nevertheless, we observed similar enhancements of lateral feature size of 40 nm Ag NPs deposited on our device after water etching for about 90 minutes. Correlated optical and AFM images in Fig. 5, demonstrate the visualization of 40 nm Ag nanoparticles using our technique. Lateral etching resulted in a spatial magnification factor of about 10 for 90 minutes of etching. This lateral magnification factor implies that a 4–5 nm particle would produce a feature size of about 40–50 nm after etching which should be readily visible in the microscope. We tried to verify this limit experimentally using spherical Au NPs and CNTs. However, the optical contrast was not sufficiently high most likely due to the lack of adhesion of these particles to the non-functionalized surface. Poor adhesion would lead to motion of the particle during the etching process and consequent reduction (wash-out) of the differential optical contrast. The results of visualization of 2 nm dia CNT is shown in the SI text [SI text, section 11]. We believe that visualization of nanoparticles or objects down to 5 nm should be possible by a) using adhesion layers to reduce fluctuations of the position of the particle during the etch process and/or b) increase the etch rate of the Ge films by increasing the GeO_2_ content during film deposition or post-deposition oxidation. Such studies are presently underway.

## Discussion

The working principle of our technique is the combination of the facts that Ge thin films on gold can produce strong color response and that GeO_2_ formed readily on the Ge film surface can be etched with water which is a benign solvent permitting imaging using water-immersion microscope objectives. The etch rate of GeO_2_ has been reported to range from 10^−2^ nm/min to 10^4^ nm/min depending on the oxidative state of Ge^27^. We have determined the etch rate in our system to be around 0.3 nm/min [SI text, section 8]. The GeO_2_ content of the Ge films prepared in our RF sputtering tool was determined to be about 28% using XPS [SI text, section 6]. Depth resolved XPS confirmed that GeO_2_ is present only near the surface and the bulk contains elemental Ge [SI text, Section 6]. This implies that the mechanism of differential etching involves both differential oxidation rates as well as differential water transport. The relative significance of these two effects must be clearly understood to use this technique for quantitative transport studies. However, the technique can be used as a nanoscopic visualization tool even in the absence of such a quantitative model. This technique also probes the heterogeneity in the water transport through nano-membranes which will be useful in the design of membranes for applications such as water desalination. One obvious limitation of this technique is that it is currently restricted to probe transport of water alone. However, by using polymer thin films which selectively etch in the presence of specific ions, it would be possible to measure the transport of these ions. For example, the solubility of PAA-Ca^2+^ increases with increasing Na^+^ concentration in water^28^. By depositing an intermediate layer or PAA-Ca^2+^ sandwiched between the nano-membrane and the Ge film, one can use this technique to probe the transport of Na^+^ ions through the nanomembrane as described in the SI text section 12. The estimated limit of detection of this technique for visualization of nanoparticles is about 5 nm which should permit single-molecule resolved studies by using functionalized NPs or QDs (4–5 nm). At this limit, it will also be possible to observe single macromolecules or polymers optically, without the use of fluorescent labels. In conclusion, we have demonstrated a nanoscopic visualization technique with significant potential in areas ranging from fundamental studies of solute transport in nanoscale membranes to single-molecule resolved sensing of molecular interactions.

## Methods

### Device Fabrication

A Si wafer was cleaned in Piranha solution (3:1 of Sulphuric acid and Hydrogen Peroxide) and used as the substrate. All thin films were deposited in a Tecport sputtering unit. A 150 nm, optically thick Au film was sputtered on the Si substrate with a 10 nm thin layer of Cr as an adhesion promoter, followed by which Ge films of thickness ranging from 5–30 nm were deposited on the Au film. Au and Ge were deposited at the rate of about 5 Å/sand 2 Å/sec respectively under a vacuum of 10^−6^ Torr. A bare Si wafer was used in addition to the metal coated substrate for ellipsometric determination of deposited Ge film thickness. In some cases the Ge film was also patterned permitting the measurement of Ge film thickness using an AFM.

### Measuring reflectance spectra from the microscope

The reflectance spectra of the devices were measured using an Ocean Optics modular fibre optic spectrometer model USB 2000. The receiving optical fibre was secured to the eye-piece of an upright microscope (Model Olympus BX51M) to collect the reflected light from the device. The reflected light collected by the optical fibre was sent to the spectrometer which was controlled by the Ocean Optics software via a USB interface. The calibrated reflectance spectra were obtained using the Ocean Optics software and the corresponding color images were simultaneously captured by a CCD (Model: Olympus DP73) attached to the camera port of the microscope.

### Synthesis and transfer of Graphene

Large area Graphene was synthesised on Cu foil in a CVD chamber with precursor gas Methane in presence of Hydrogen. All the graphene films were grown at a temperature of 1000 °C under a total pressure of 4 Torr. Graphene grown on the Cu foil was transferred on to Ge thin film devices using the conventional wet transfer process. The graphene-Cu composite was initially coated with a thin PMMA support layer followed by a soft bake at 180 °C. The underlying Cu was subsequently etched using ammonium persulphate solution. The free standing graphene-PMMA composite was then fished out using an oxidized Si wafer. After rinsing with de-ionised water for a few times, the graphene layer was finally transferred on to the Ge thin film devices. The final graphene-on-Ge film was then vacuum dried using a desiccator for 3 hours to avoid trapped water between graphene and the Ge thin film surface.

Further details of the experimental protocols and characterization procedures are provided in the supplementary document, SI text sections 1–7.

## Additional Information

**How to cite this article**: Suran, S. *et al*. Bright-field Nanoscopy: Visualizing Nano-structures with Localized Optical Contrast Using a Conventional Microscope. *Sci. Rep*. **6**, 25011; doi: 10.1038/srep25011 (2016).

## Supplementary Material

Supplementary Information

Supplementary Video S1

Supplementary Video S2
