# Supplementary material for: Bright-field Nanoscopy: Visualizing Nano-structures with Localized Optical Contrast Using a Conventional Microscope
Source: Sci Rep. 2016 Apr 26;6:25011. doi: 10.1038/srep25011 (PMC4845020; doi:10.1038/srep25011)
Supplement: Supplementary Information [file srep25011-s1.pdf]

SUPPORTING INFORMATION

Bright-field Nanoscopy: Visualizing Nano-structures with Localized Optical Contrast Using a Conventional Microscope

Swathi Suran,[a] Krishna Bharadwaj,[a] Srinivasan Raghavan [a] and Manoj M. Varma [a,b,*]

[a] Center for Nano Science and Engineering (CeNSE), Indian Institute of Science

[b] Electrical Communication Engineering, Indian Institute of Science

* Email: mvarma@cense.iisc.ernet.in

Section 1: Fabrication and characterization of the device

A p-type <100> oriented silicon wafer was cleaned in piranha solution consisting of 3 parts of sulphuric acid and 1 part of hydrogen peroxide. The wafer was bathed in buffered hydrofluoric acid (BHF) to remove any native oxide present on the surface. Subsequently, chromium and gold films of thicknesses 10 nm and 150 nm respectively were deposited in a Tecport sputter coater. Chromium was sputtered to improve the adhesion of the gold to the Si substrate. No substrate heating was used during sputtering. Au was deposited at a rate of 5Å/sec under high vacuum of 10^{-6}Torr using DC power. Followed by Au, germanium was sputter deposited at a rate of 2Å/sec using RF power to attain different thickness varying from 5-30nm by changing the deposition time. For thickness optimisation, circular patterns of 100 and 200 µm were patterned by photolithography in EVG 620 double sided mask aligner using a dark field mask. Subsequently, germanium of different thicknesses was sputtered on these lithographically patterned substrates after which the photoresist was stripped off leaving Ge spots of 100 and 200µm diameter. The thickness of these patterns was measured in a Bruker Scan Asyst Atomic Force Microscope in the tapping mode. Parallally, Ge films were also deposited under exactly similar sputtering conditions on a bare Si wafer so that the thickness of Ge could also be measured optically using a Variable Angle Spectroscopic Ellipsometer (VASE) J. A. Woollam Co. M2000U. From Figure S1., it is seen that the thickness of Ge measured using Ellipsometry and AFM are in reasonable agreement.

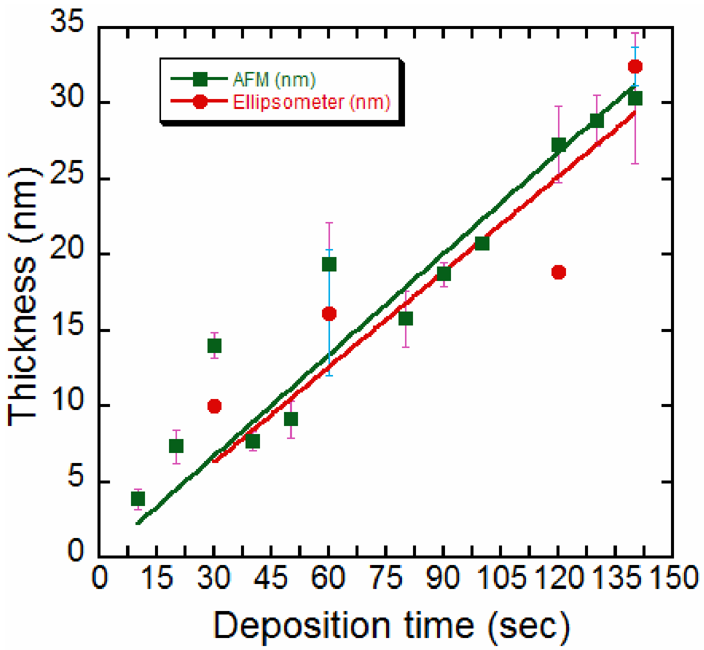

Figure S1. Correlation between Ellipsometer and AFM measurements of the thickness of Ge film deposited for different deposition times.

Section 2: Reflectance spectrum measurement

Different thicknesses of Ge on Au, give rise to an array of uniquely separated colors which were captured using an upright microscope from Olympus, BX51M in the bright-field mode. The color response is due to interference of the reflected light from the top and bottom interfaces of the Ge film. In order to measure the reflectance spectra, we used a table-top, miniature fibre-optic spectrometer by Ocean Optics, the USB2000 model. We mounted the receiving optical fibre of the spectrometer on the optical microscope such that it collect all the reflected light from sample through the eye-piece. The light from the eye-piece was carefully focused to the inlet of the optical fibre. The spectrometer was connected to the microscope's computer via the USB port. This set up enabled us to capture both the reflectance spectra and bright field images of the device simultaneously. The bright field images of the different thicknesses of Ge deposited on Au are shown in Fig 1(d) in the main text. All images were captured in the illumination from the Halogen lamp without any external filters.With increasing thickness of Ge, we observe a red-shift of the reflectance spectra, as expected [1].

Section 3: Theoretical calculation of reflectance spectra

We used a transfer matrix method to calculate the reflectance spectrum of the Ge thin film structure shown in Fig. 1 (c) of the main text [2]. The calculations were done using the MATLAB software package. The refractive index for Si and Au used in the simulation were obtained from an online RI data base [3], whereas the optical constants used for Ge were extracted from the ellipsometer data for a 30 nm thick sputtered Ge film. As seen from Fig. 1 (b) and (c) of the main text, the calculated spectra are in good qualitative agreement with the experimentally measured reflectance spectra.

Section 4: Estimating etch rates of sputtered germanium thin films in water

Ge film readily oxidises itself to become GeO_2 which is soluble in water [4]. The oxidation and consequently the oxide film is confined to the surface. All our experiments were carried out within 2-3 days from the time of deposition. Assuming that these films etch isotropically with water, we estimated the etch rates and discuss its association with the reflectance spectra in this section. Five different thicknesses of Ge, namely, 14, 20, 22, 26 and 30nm, were deposited on Au substrates in the array pattern described in section 1. The initial thicknesses were measured in an AFM. These samples were then etched in DI water for a duration of 15 mins and the thickness of the Ge layer was measured again using the AFM. The average etch rate observed for all these samples was about 0.3 nm /min.

A further study was performed where different devices, all with the same initial thickness, were etched for different durations. The height difference as a function of etch time was plotted as shown in Fig. S2. This study also yielded an average etch rate of about 0.3 nm/min. The reflectance spectra were captured as a function of time as the films underwent progressive etching in water. With the spectrometer mounted on the microscope, we were able to simultaneously record both the optical image as well as reflectance spectra. When recording the reflectance spectra from Ge patternsthe Field Stop (FS) of the microscope was adjusted such that only the light reflecting from the Ge region is collected by the spectrometer's optical fibre. The reflectance spectra as a function of etch time is shown in Fig. S3. It can be seen from Fig. S4 that the spectral shift due to Ge thickness change is about 13 nm/nm, i.e. 13 nm shift in wavelength per nm of change in Ge thickness. Given that even crude spectrometers can easily achieve nm

level resolution of spectral features, this means that angstrom level changes in Ge film thickness due to etching can be quite easily measured. Further, we note from Fig. S3, that the slope of the spectral shift curve (13 nm/nm) was fairly robust across several samples.

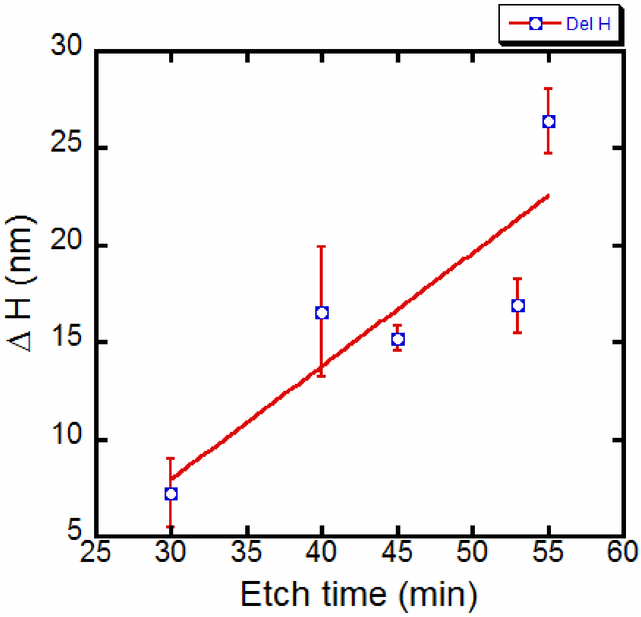

Etch Time ΔT	Average ΔH	Error
55 min	26.39842	1.682303
53 min	16.89184	1.385713
45 min	15.2	0.653545
40 min	16.54216	3.335771
30 min	7.21833	1.774665

Figure S2: Change in Ge film thickness as a function of etch time.

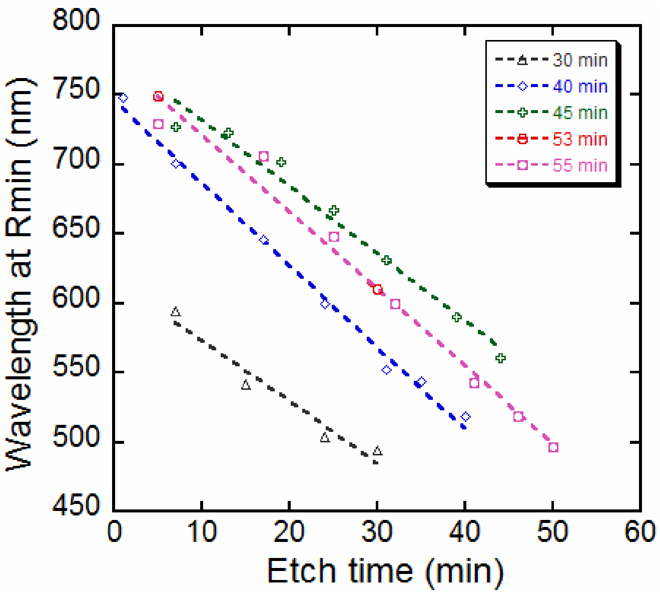

Figure S3: Spectral shift (shift of the position of minimum reflectance) of the Ge devices as a function of etch time in DI water

Section 5: Mechanism of visualisation due to differential etch

Ready oxidation of Germanium to GeO_2 and its dissolution in water and the capability of the device to pick up small changes in the Ge thickness [1] with a high contrast enabled the visualisation of nano-structures. Any nano-structure like nano-particles or graphene with defects on the device when underwent water etch, as shown in the Fig. S4 a. & b. manifested to local differences in etch of the underlying Ge which was easily visualized using a BF optical microscope with a large color contrast.

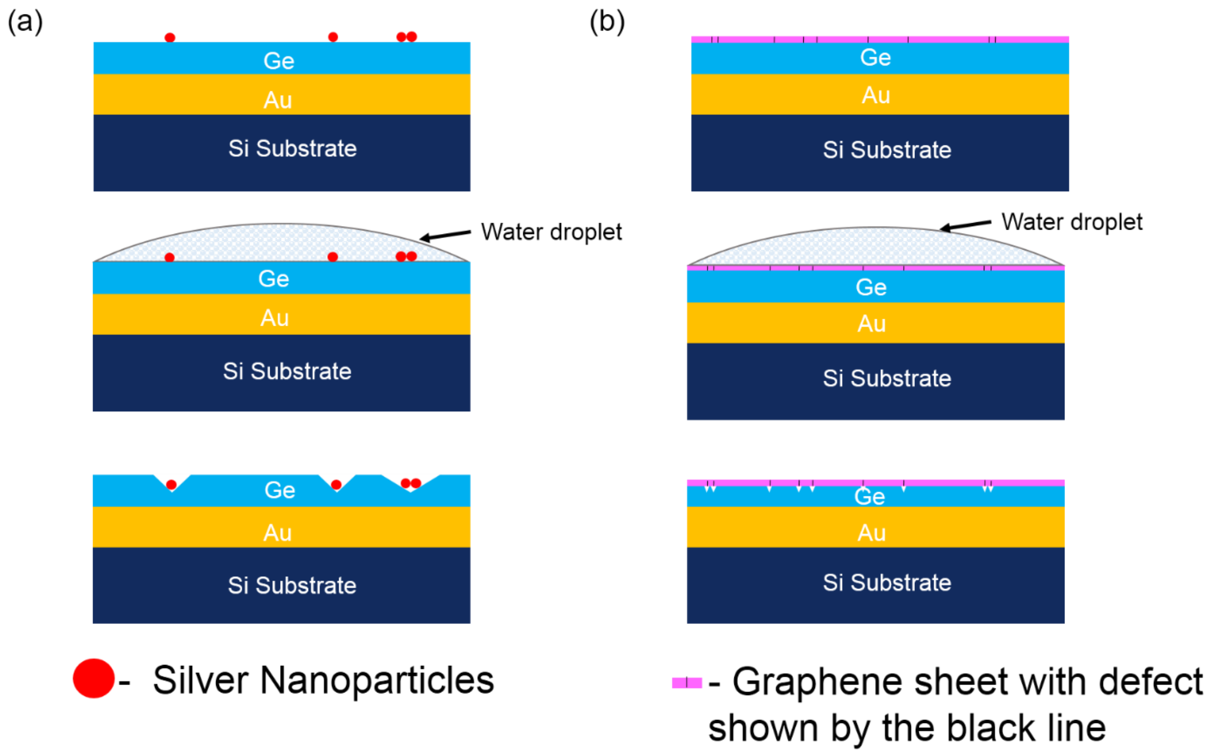

Figure S4: a. Ag NPs drop casted on the device undergoes etch in the presence of water, resulting in anisotropically etched pits in Ge. **b.** Graphene placed on the device. On water etching, the Ge beneath the GGBs (marked in black) etch faster when compared to the rest of the area (grains).

Section 6: Analysis of native oxide on Ge using XPS

As our technique strongly depends on the oxide content in the Ge film, we carried out studies using X-ray Photoelectron Spectroscopy (XPS, Model: Axis Ultra) to determine the composition of native oxide in the sputtered Ge films. Fig. S5 (a) shows the Ge 3d high resolution spectrum which indicates the presence of two oxidation states of Ge. The composition was estimated to be 49.2% Ge, 22.5% GeO and 28% GeO_2. The experimental plot in the Ge 3d spectrum comprises of two major peaks as seen in Fig. S5 (a). The large peak is associated to elemental Ge at 29.3 eV and deconvolution (peak-fitting) shows the presence of Ge in two different oxidation states, GeO (+2) at 30.9 eV and GeO_2 (+4) at 32.5 eV. Further, depth-resolved XPS was carried out to determine the oxide present at the surface and how much it extends through the depth of the film. Depth resolved XPS revealed that the oxide (GeO_2) is confined to few nanometer thickness from the top surface of Ge. Fig S5 (b) shows Ge 2p $_{3/2}$ component of the Ge 2p high resolution spectrum. The Ge 2p before etch spectrum shows a significant presence of Oxygen as a second peak at 1220.4 eV other than the peak due to elemental Ge at 1217 eV. With consecutive etches, it was seen that the intensity of the peak at 1220.4 eV diminished as the Oxygen content decayed through the thickness of Ge. This implies that the mechanism of etching involves oxidation of Ge to GeO_2 due to dissolved O_2 content in the water followed by dissolution of GeO_2 due to water. Further studies are underway to control the GeO_2 content of the film across the depth to achieve etch rates of the order of 5 nm/min. The peak-fitting and other analysis of Ge thin film was carried out using the open-source software available online, CasaXPS [5].

(a)

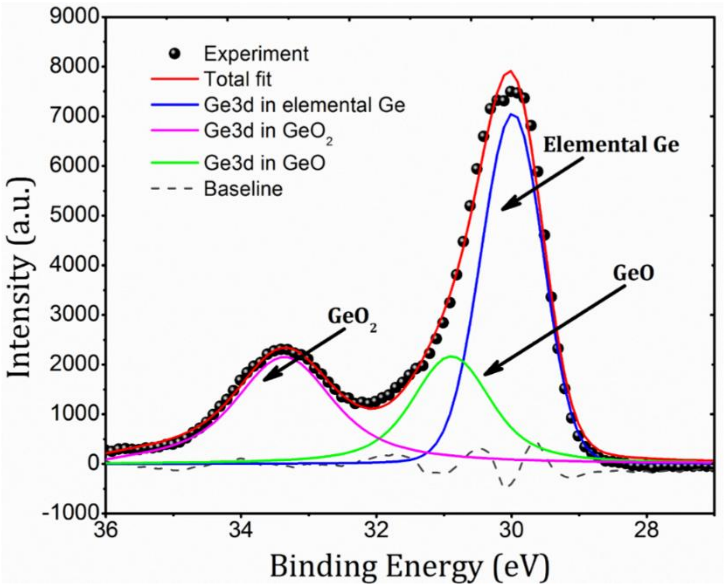

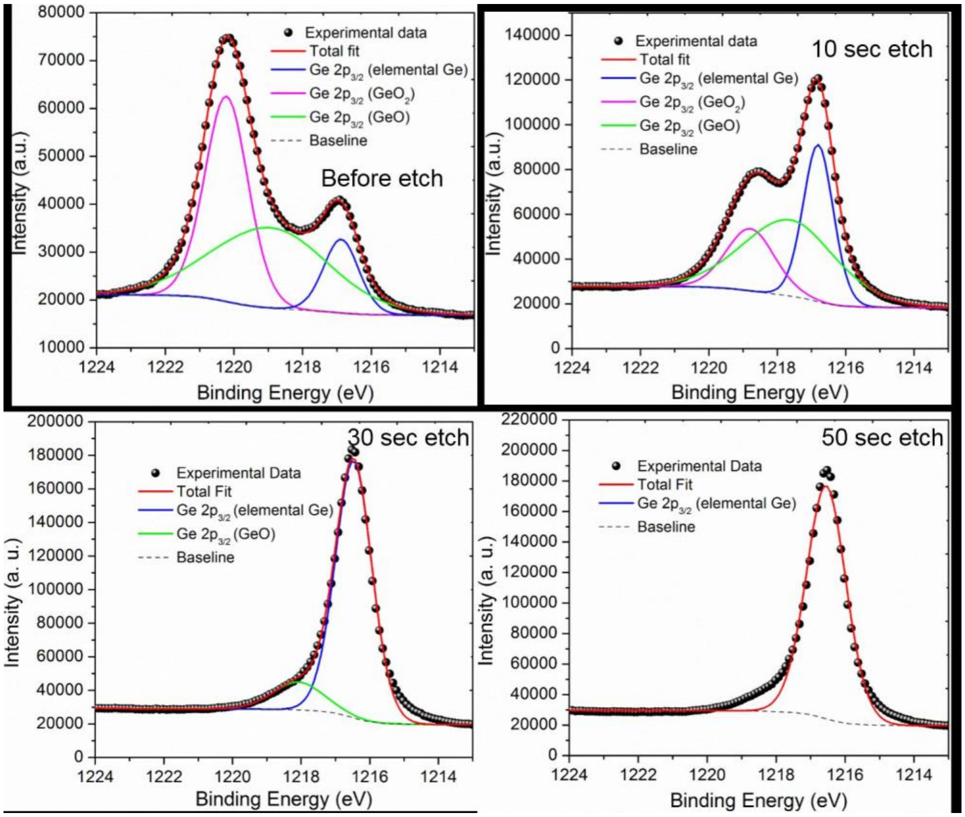

Figure S5: a. High resolution XPS spectrum of Ge in the $3d_{5/2}$ energy region showing the presence of native oxide on Ge. **b.** Depth resolved XPS showing the decay of oxygen content through the thickness of Ge. At the end of 30 sec etch cycle, the composition of the film was 100% elemental Ge.

Section 7: Raman and SEM measurements of graphene layer on germanium

Figure S6 shows the typical Raman spectrum from the single layer graphene layers transferred on to the Ge film. The Raman spectra were obtained using a 535 nm laser in a Horiba LabRAM HR. Three important peaks are noted in the figure as D (1350 cm^{-1}), G (1580 cm^{-1}) and the 2D (2750 cm^{-1}). While the 'D' intensity is quantitative measure of the defect density in graphene under the laser, the 2D peak width is an indicator of the number of layers. Low relative 'D' peak intensity ($I_D/I_G < 0.2$ and $I_{2D}/I_G > 3$) and a small 2D peak width (FWHM of 2D < 30 cm^{-1}) confirm the presence of good quality single layer graphene. Raman measurements were made before and after the DI water etching experiments to check if the etching caused any significant

change in the graphene layer. Raman data shown in Fig. S7 confirms the presence of graphene after the etch experiments and additionally, no significant difference in the 'D' peak intensity ratio, $\Delta\ I_D/I_G < 0.1$ indicates that the graphene is not significantly modified by the etching process.

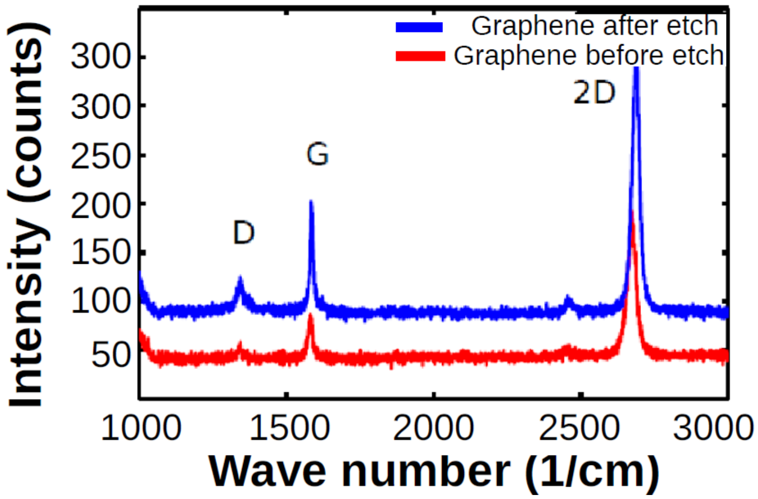

Figure S6: Raman spectra from single layer graphene transferred on the Ge thin film device. The spectra were obtained before and after the DI water etching of the underlying Ge film.

An estimate of the graphene grain size was obtained by analyzing the scanning electron microscopic (SEM) images of the grown grapheme just before coalescence of the film. Figure S7 shows a representative SEM image indicating that the graphene grains are mostly hexagonal with sides of length of 2-5 µm.

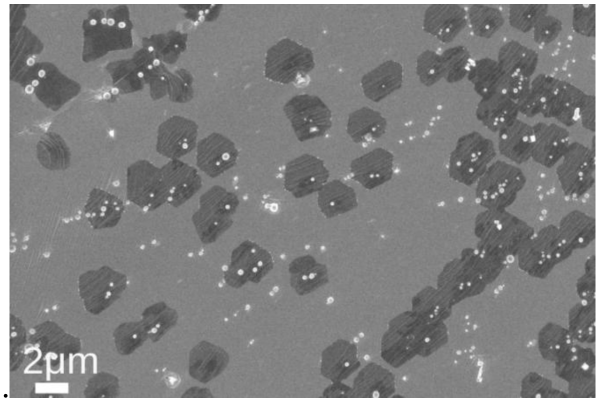

Figure S7: SEM image of Cu surface showing graphene grains before coalescence. Grains are mostly hexagonal shaped with sides of length 2-5 µm.

Section 8: Original/ raw data of the images produced in the main text.

Figure 2 in the main text illustrates the first observation of GGBs and also other anomalies in the transferred graphene. The images in the main text were obtained after changing the color balance of the raw image for optimal visual detection of the features. The original images before adjusting the color balance are provided in this section. The color balance of the raw images where adjusted using the Adjust – color balance option in the open source software package ImageJ. The raw and color balance adjusted images are shown side by side in Fig. S8 below.

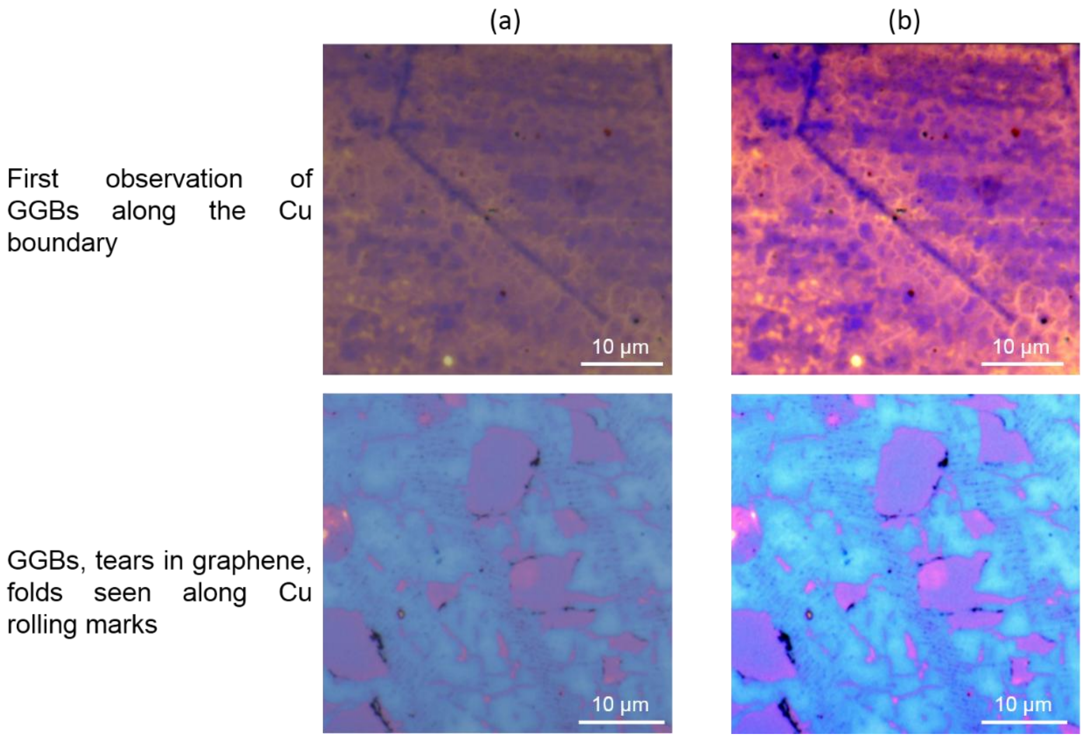

Figure S8: Post processed raw images. a. Raw and **b.** color balance adjusted images where grain boundaries and other defects are seen in the single layer graphene.

In Fig. S9 below, the encircled area in the time sequence image set in Fig 2 (d) from the main text has been magnified for better visualisation of the grain boundaries. Note that these images have not undergone any color balance adjustment. The grain boundaries have been marked with red solid lines to delineate them.

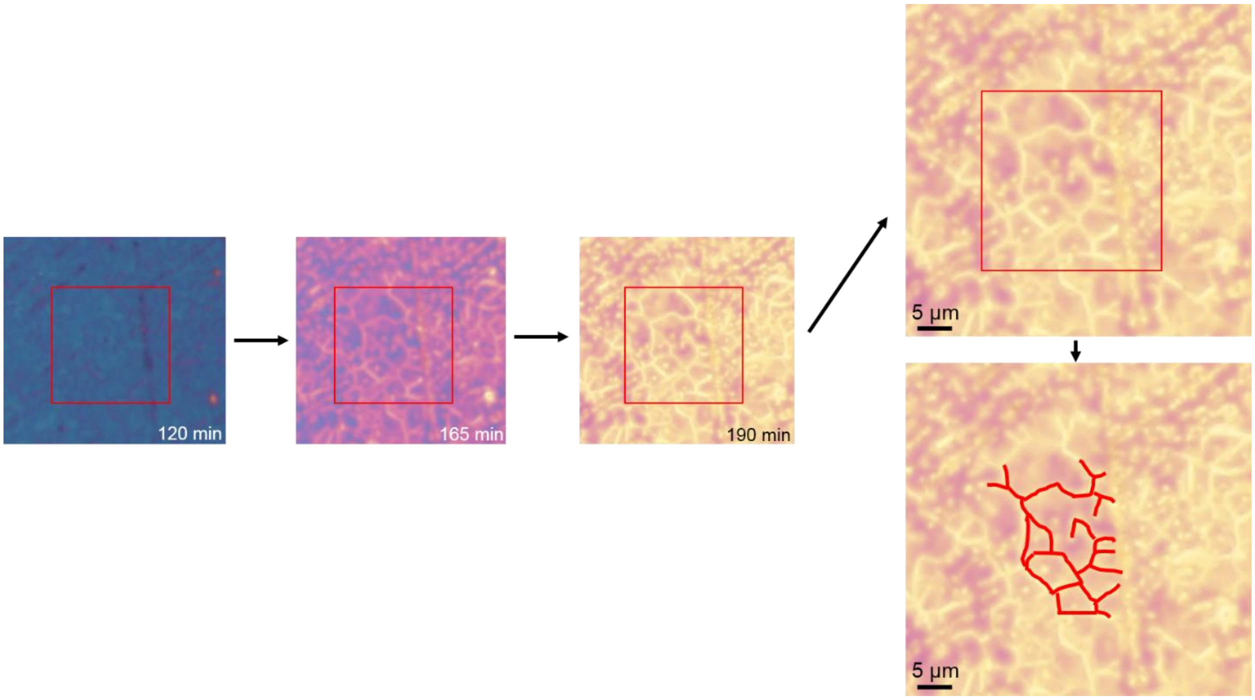

Figure S9: Magnified images of the region with graphene grain boundaries (Magnified from Fig. 2 (d) of the main text.

As mentioned in the main manuscript, Differential Interference Contrast (DIC) imaging mode provided better optical contrast compared to Bright-field imaging mode. Fig. 3 in the main text shows the raw BF images as well as DIC images after adjusting color balance. Fig. S10 below shows the raw DIC images and the comparison of raw BF and DIC imaging modes.

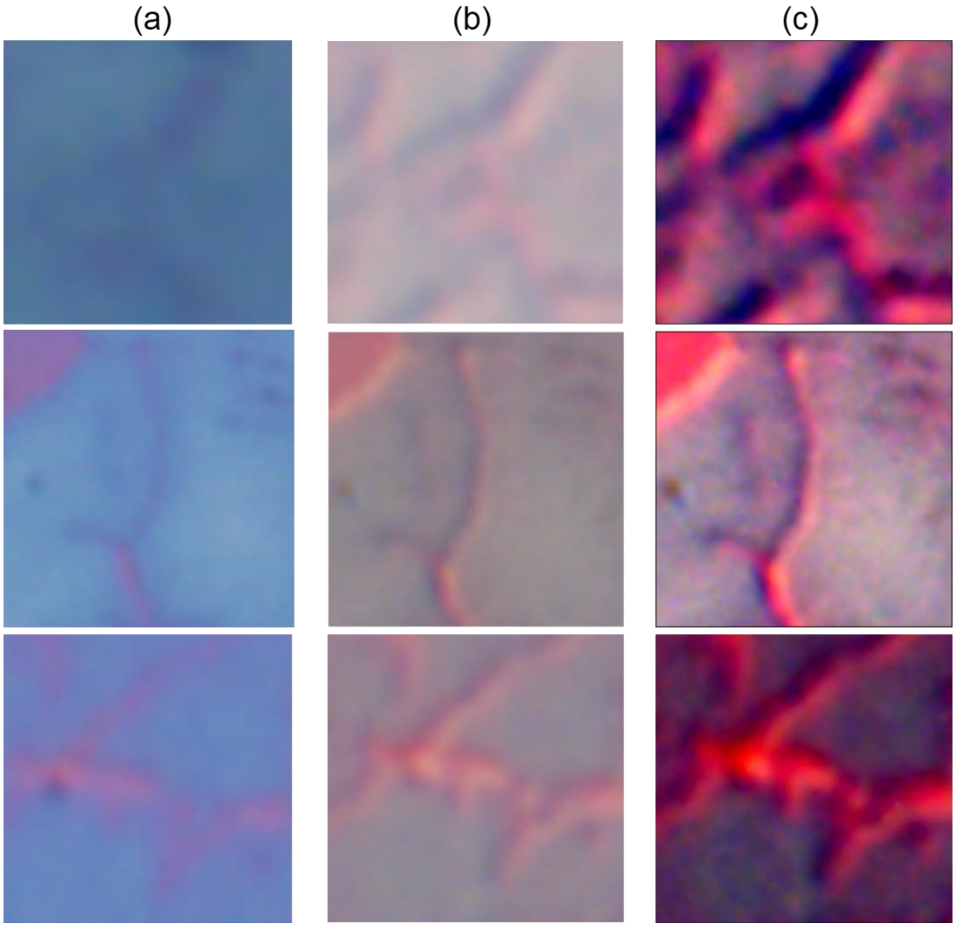

Figure S10: Comparison of raw BF and DIC imaging modes for nanoscopy. **a.** Raw BF images where GGBs from graphene grown under different growth conditions. **b.** Raw DIC images from the same location as in **a. b.** Processed (color-balance corrected) images showing improved visualisation of GGBs.

SVideo 1: Time course of water transport through GGBs

Section 9: Differential water transport through ultra-thin polymer layer

Polymer nano-membranes (single layer of PAH, ~1-2nm, as described in the main text) patterned on 30 nm Ge devices became visible with increasing optical contrast as the differential etching of the Ge film progressed. We measured the step height due to polymer to be around 1 nm before the beginning of the etch process. After 90 minutes of etch the step height increased to about 2.74 nm when measured in AFM as seen in Fig. S11. The increase in height is because of

the Ge film directly underneath the polymer layer not etching as fast as the background, as the polymer layer impedes the access of the etchant (water) to the Ge film. From these height differences, we can estimate that the etch rate of background Ge (not under the polymer) is about 0.333 nm/min whereas etch rate of Ge through the polymer is 0.311 nm/min. The etching of Ge is approximately 6% slower through a single layer of polymer measuring about 1 nm in thickness. From this data, we estimated the volume flow rate of water through the polymer in the following manner. Let τ_p be the time it takes to etch one nm of Ge film directly under the polymer. Similarly, τ_b is the time for one nm of Ge film directly exposed to water to etch. Then we wrote τ_p as,

$$\tau_p = \tau_b + \tau_w$$

Where, τ_w is the time taken by water to permeate through the polymer layer. We estimated τ_p and τ_b from the inverse of the etch rates 0.31 nm/min and 0.33 nm/min respectively yielding τ_w to be about 0.19 minutes. This is the time it takes water to permeate a roughly 1 nm thkck polymer layer. Therefore the rate of water permeation is about 1 nm/0.19 minutes or about 5.1 nm/min. This is equivalent to a volume flow rate of 510 pL/min. It is quite obvious that this estimate is not a very precise one due to the uncertainties and variations in step heights between various polymer pillars. A precise quantitative estimate will require a rigorous model of color change or spectral shift as a function of water transport rate through membranes. Such a model should account for change in Oxygen permeability due to the nanomembrane as well as water transport through the membrane. However, in the present work we employed the crude model described above to illustrate the potential of technique in transport measurements through nano-membranes.

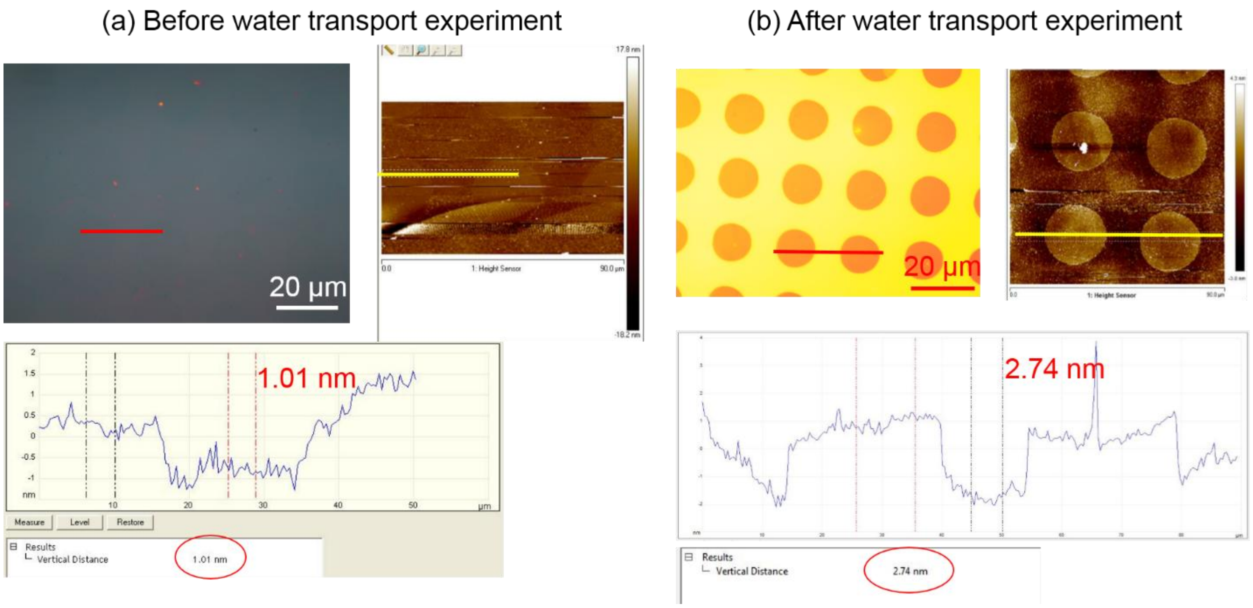

Figure S11: AFM measurements taken before and after experiments of water transport through polymer nanomembrane. a. shows the optical image of the polymer NM patterned on a 30nm Ge device which is invisible at the start of the etch. The thickness of the polymer measured is 1.01 nm. **b.** shows optical image of the polymer NM patterns clearly visible at the end of the etching process. The AFM measurement shows a total thickness of 2.74 nm measured across the patterns, which includes the 1 nm thick polymer and the height difference in Ge film due to the differential etch rate under the polymer.

SVideo 2 shows the complete time course of the etching process. Some snapshots from the video are provided in Fig. S12 below.

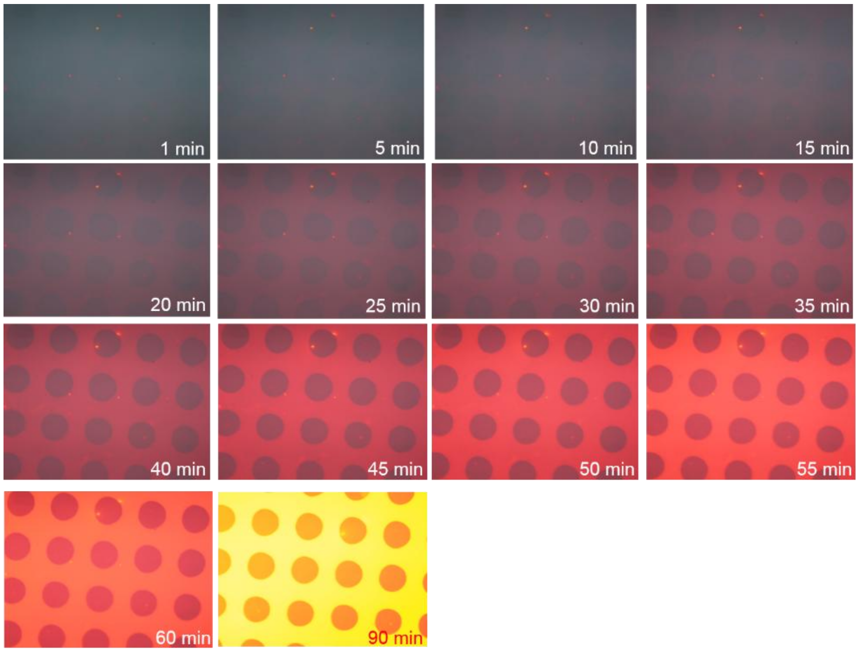

Figure S12: Time course of water transport through a single layer polymer of 1 nm thickness. The time at which each image was captured is indicated below in each of the images.

Section 10: Scanning Electron Microscope (SEM) images of Ag NPs

For the visualisation of nanoparticles on Ge devices using our technique, we used silver (Ag) nanoplatesof lateral dimension around 40 nm from Nanocomposix Inc. The concentration of Ag NPs used was 21 x 10^{-14}gm/mL (10^9 Ag NPs/ mL) in DI water with a pH maintained at 9.3. The SEM image of the Ag NPs dispersed on a Piranha cleaned bare Si wafer (Fig. S13) confirms the size of single NPs around 40 nm. The SEM used was Zeiss Gemini SEM model.

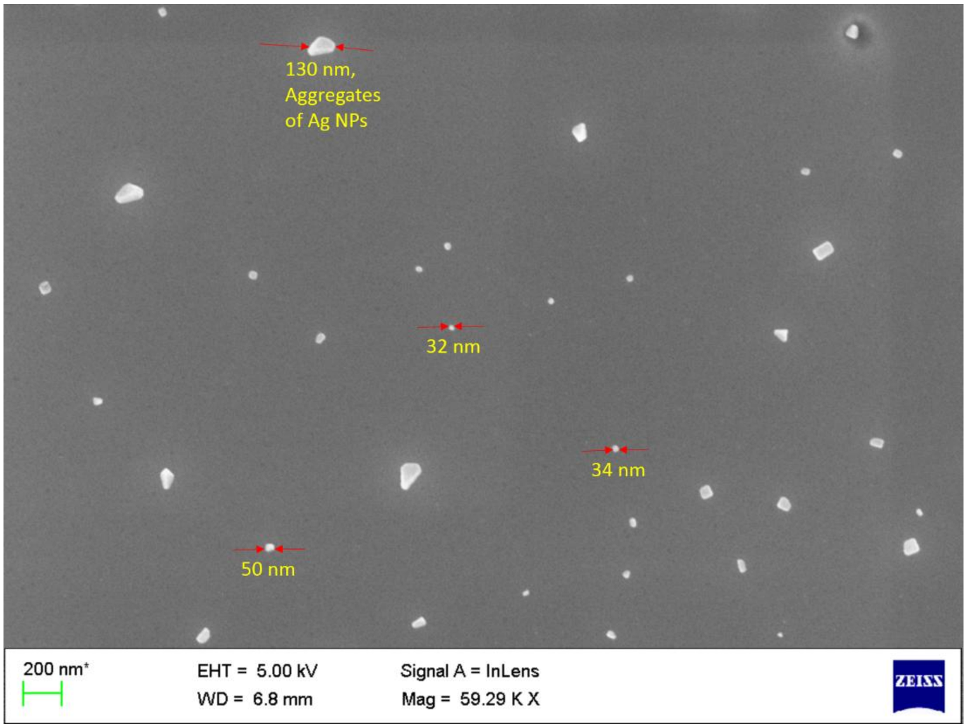

Figure S13: Electron Microscope image of Ag NPs drop casted on Silicon.

Section 11: Visualization of single CNTs using differential etching

In order to probe the ultimate limit of detection/visualization using this technique, we attempted to visualize Single Walled Carbon Nano Tubes (SW-CNTs). A solution of SW-CNTs with concentration 10ng/ mL containing CNTs with diameter 1-2 nm and length 2-3 microns was used. A 10 µL of this solution was drop casted on the device and left to dry at room temperature so that the SW-CNTs settle and adhere to the Ge device. Water etching experiments carried out on these samples revealed certain features in the Bright-field optical images correlated with Atomic Force Microscopy (AFM) images to be single SW-CNTs (Fig. S14 (a)). Figure S14 (b) and (c) are BF images after image processing where two well separated CNTs are identified while (d) and (e) are the AFM 2D scan and height profile across the CNT marked in (c) and (d). The location of the SW-CNTs are indicated by the red solid lines in Fig S 14 (c) and yellow solid lines in Fig S 14 (d).The optical contrast was quite poor in these experiments. We believe that the poor contrast could be due to movement of CNTs during the rather long etching process. Spatial movement of the CNTs will cause the contrast to get averaged out. Improving the

adhesion of the CNTs to the surface using functionalization layer and/or modifying the Ge film to achieve much faster etch rates is likely to improve the optical contrast.

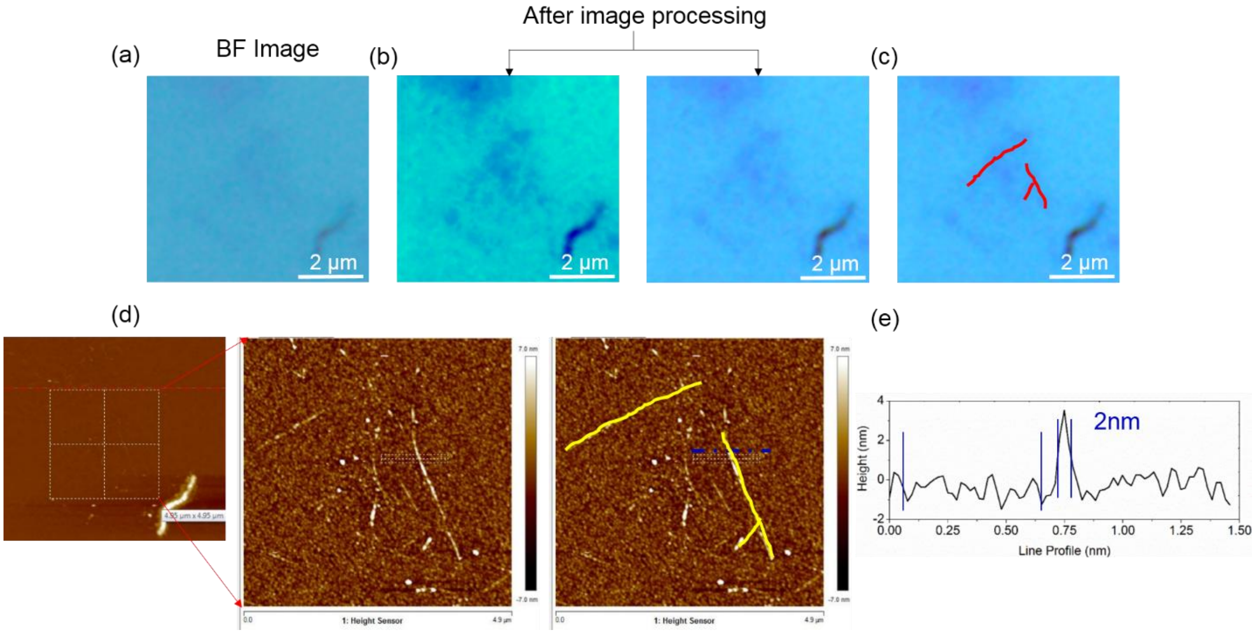

Figure S14: Visualizing single CNTs. a. Optical image captured in the BF mode. **b.** and **c.** BF image in **a.** after image processing. The location of the isolated CNTs are indicated by the red solid lines drawn over them. **d.** 2D AFM map of the corresponding location in **a.e.** Height profile indicating the CNT's height measured across the blue dotted line in **d.**

Section 12: Measuring transport of ions using ion-selective layers

As mentioned in the discussion section of the main text, one obvious limitation of this technique is that it is currently restricted to probe transport of water alone. However, by using polymer thin films which selectively etch in the presence of specific ions, it will be possible to measure the transport of these ions. As an example, consider the measurement of Na+ ion transport through 2D membranes. In this case, Ca^{2+} conjugated poly-acrylic acid (PAA-Ca^{2+}) can act as an ion selective layer as the solubility of PAA-Ca^{2+} increases with increasing Na^{+} concentration in water [Ref. 28 in the main text]. By depositing an intermediate layer or PAA-Ca^{2+} sandwiched between the nano-membrane and the Ge film as shown in Fig. S15 below, one can probe the transport of Na^{+} ions through the nanomembrane. For the film structure shown in Fig. S15, consider that the nanomembrane impedes the transport of Na^{+} ions. In that case the ion selective

$PAA\text{-}Ca^{2+}$ layer would dissolve at a faster rate outside the area covered by the membrane leading to a differential etch rate and Ge film thickness difference as shown in Fig. S15. The relative color difference between the area covered by the membrane and the background is a measure of Na^{+} ion transport through the membrane. Quantitative models can then be employed to extract relevant transport parameters.

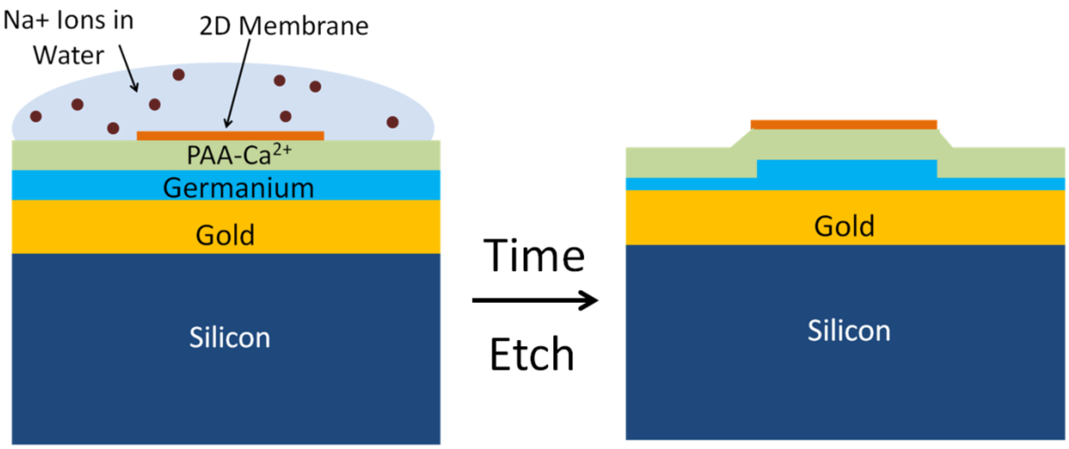

Fig. S15: Schematic illustration of measuring Na+ ion transport using an ion selective $PAA\text{-}Ca^{2+}$ intermediate layer.

REFERENCES

1. Kats, M., *et al*., Nanometre optical coatings based on strong interference effects in highly absorbing media. *Nat.Mat* Vol. **12** (2013).

2. Born, M & Wolf, E *Principles of Optics* 7th edn (Cambridge Univ. Press, 2003).

3. Refractive Index online database: http://refractiveindex.info/.

4. Kawase, T. *et al*. Metal-assisted chemical etching of Ge (100) surfaces in water toward nanoscale patterning. *Nanoscale Research Lett*, **8:**151, 1-7 (2014).

5. http://www.casaxps.com/
